# A Critical Review of Global Digital Divide and the Role of Technology in Healthcare

**DOI:** 10.7759/cureus.29739

**Published:** 2022-09-29

**Authors:** Himabindu Reddy, Shiv Joshi, Abhishek Joshi, Vasant Wagh

**Affiliations:** 1 Community Medicine, Jawaharlal Nehru Medical College, Datta Meghe Institute of Medical Sciences, Wardha, IND

**Keywords:** healthcare technology, inequity, digital health, e-health, healthcare access, digital divide

## Abstract

Healthcare and technology, the fusion of these two distinct sciences can be traced back to the Vedic era. Regrettably, while it is evident that the journey of advancements in knowledge and innovation leading to the advent of technology to better the health of mankind is not a recent one, owing to inexistent means of transfer of knowledge, these contraptions stayed mostly localized to the regions of their inventors. This article seeks to review the vital role that technology has in bettering the health status of the global community and the challenges associated with healthcare technologies like inequity in connectivity, affordability, and accessibility. Technology and artificial intelligence are integrated to the best of the health systems across the world but these advancements are not accessible to a considerable part of the global population. While affordability, the absence of a steady internet supply, and the lack of a device to use the technology are the major impediments causing this digital divide, cultural factors and health literacy also contribute to this scenario. Nevertheless, access to the internet has been recognized as a basic need by all governments around the globe. The COVID-19 pandemic shook the health systems of developed and developing countries alike and has made every administration feel the urgency in making healthcare more accessible. Having seamless internet coverage and setups to make telemedicine or online consultations possible, can contribute significantly in paving the path to making our societies prosperous and healthier. With the world’s consensus about this goal, efforts now should be focused on research and development for making these technologies more affordable and accessible without compromising their utility.

## Introduction and background

Healthcare and healthcare systems constitute an intricate nexus involving players from public-private, regional-central, and national-international sectors, and care must be taken to ensure effective collaborations of all these players. Today's world and technological progress go hand in hand but despite all the advanced mechanisms available to us, the pandemic led to jarring health outcomes in developed and developing nations alike. The world and its health systems are grappling with the repercussions of COVID-19 and the need of the hour is to develop more affordable technology and build sustainable and resilient health care systems. Effective medical technology, and health policies focusing on ensuring equitable access to medicines, along with health technologies that aid in leading a healthy and productive life, form the three pillars of a robust healthcare system [1].

Health technology as defined by World Health Organization (WHO) is "the set of techniques, drugs, equipment, and procedures used by health care professionals in delivering medical care to individuals and the system within which such care is delivered.” It alludes to all types of technology that are used to amp up the efficacy of treatments and better delivery of medical and surgical sciences [2]. Healthcare and technology, the fusion of these two distinct sciences can be traced back to the Vedic era of Sushruta, the father of plastic surgery. In his annals of surgical techniques, the 'Sushruta Samhita', he elaborated on the use of over 100 surgical instruments, besides the use of non-invasive techniques of beams of light and heat in healing techniques. His inventive innovations in delivering healthcare solutions ranging from trichology and plastic surgery to repairing orthopedic injuries and postoperative physiotherapy are a baffling treatise to healthcare professionals. He also pioneered the procedure of rhinoplasty which is still in practice as the Indian flap procedure [3]. Another remarkable example of healthcare-related technology in world history can be the practices of ancient Egyptians who weaved science into their culture and left behind mummies with heliographs that tell of neurosurgeries performed in that era. Archaeological evidence of cranial trepanning was found across Europe and while the drilling techniques seem to vary, the success rates of these ancient surgical techniques throw light on the potential technology available to the healthcare workers then [4].

Regrettably, while it is evident that the journey of advancements in knowledge and innovation leading to the advent of technology to better the health of mankind is not recent, owing to inexistent means of transfer of knowledge, these contraptions stayed mostly localized to the regions of their inventors. But that is not the case now. Technology today is shaping the way forward in health sciences. We now have access to an updated knowledge base that includes lessons from our history. The greatest achievements in public health like the worldwide eradication of smallpox, elimination of polio, and control of communicable diseases like malaria, rubella, etc. are the results of effective use of appropriate technology [5]. The steadily increasing prevalence rates of lifestyle diseases, ubiquitous health inequalities, and an expanding geriatric populace drive the need for more appropriate health-related technology that is accessible, affordable, reliable, and efficient in improving the health profiles of citizens. With the advent of integrating artificial intelligence (AI) in nanotechnology, particularly in nanomedicine and disease surveillance, there now is an increased scope and hope of bringing about equitable access and uniform health services delivery across the population regardless of one's social and economic strata. Major specialties like oncology, cardiology, and neurology have embraced AI tools to help with their scope of services [6].

This era is one of widespread sharing of resources, technological innovations, and information beyond borders. The spread relies on the quality production of low-cost diagnostic kits for disease screening, making essential vaccines available for all, developing sound systems of storage of drugs and vaccine reagents, and creating awareness about diseases and health using the multitude of media platforms. Recently, as the world struggled to cope with the pandemic, every piece of appropriate or innovative technology being invented or uncovered was being shared to maximize health benefits and minimize loss of life. Organizations across the globe strived to make available technology more accessible and ensure that resources reached the ground where they were needed the most. Owing to the prevalent socio-economic, cultural, financial, and other quality of healthcare determining disparities, the potential of health technology to increase access and achieve better health outcomes was not been fully materialized [7]. Lockdowns and consequent changes in lifestyles during the recent pandemic also highlighted and brought to focus the issue of inequity in digital healthcare access across the globe. The concept of the digital divide, first conceived in 1995 refers to the divide between the population which can use and have access to digital media-associated technologies and those bereft from it [8]. In 2020, A.Guterres, then the United Nations secretary general expressed his opinion on digital technology in healthcare calling it the centerpiece to every facet of response to COVID-19 challenges, be it online schooling, e-commerce, telehealth, or vaccine development and research. Furthermore, he remarked upon the global digital divide, labeling it a matter of life and death for the populace deprived of healthcare information and services [9].

This article seeks to review the vital role that technology has in bettering the health status of the global community and the challenges associated with the global digital divide in healthcare access.

## Review

**Figure 1:**
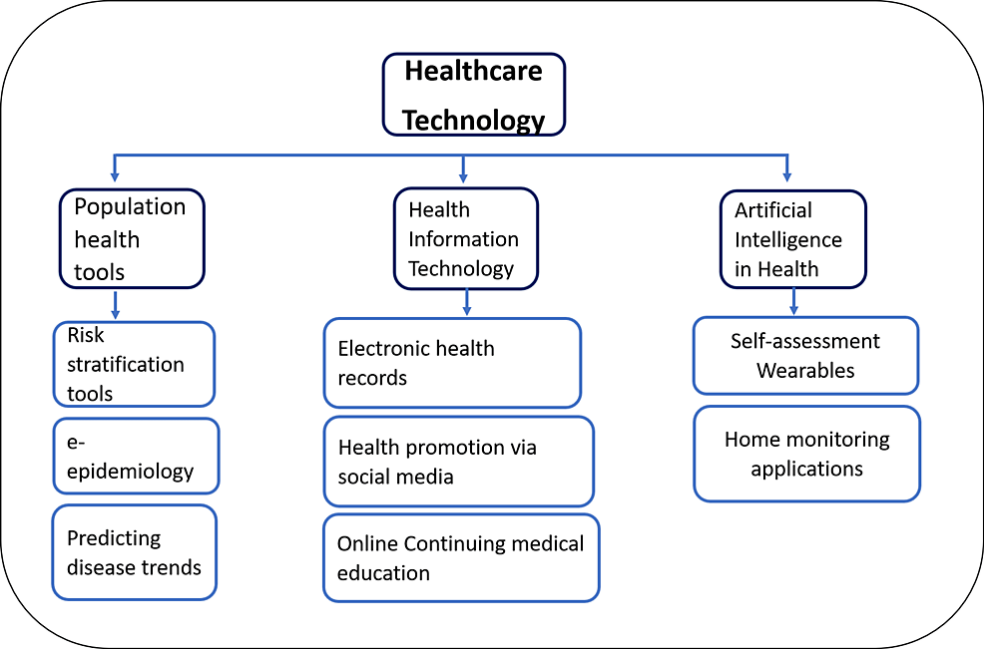
Various roles of technology in healthcare

Healthcare technology has many facets and functions in today’s world. Figure 1 demonstrates a few avenues of healthcare technology in a tree diagram.

Disruptive innovation

Innovation can be widely categorized as radical, incremental, and disruptive [10]. Disruptive innovation is a term used to describe the process by which a smaller company with fewer resources can successfully challenge established incumbent businesses. The term 'disruptive innovation' came about in a 1995 paper by Clayton-Christensen et al, who modified their description of the term in 2015 to clarify that a disruptor can be specifically labeled so only retrospectively. The entry of ‘disruptive innovation’ into clinical sciences is very recent [11]. Owing to the lack of a healthcare-specific description of this term, dubiety clouds the accurate usage of this term. This ambiguity in turn muddles up the chances of stakeholders and policymakers acknowledging these innovations as an opportunity at the earliest [12]. Some examples of disruptive innovation in healthcare include the invention of wearable health trackers, home health monitors, etc., which are discussed later in the review. A dearth of comparison studies involving two innovations, and expert-reviewed literature identifying successful applications leads to many major opportune innovations remaining hidden and obscure from the people who might benefit from their use [13]. Understanding these broken links helps build on our understanding of the chain of challenges faced by many healthcare technologies in their journey from conception to being widely accessible to the target population. This is a pivotal point of concern for health tech delivery strategies in the making and should receive due consideration.

Population health tools

Population health encompasses the multitude of health outcomes of all groups of the population along with their respective distribution within the population. The various groups within a population can be classified based on nationality, race, cultural variations, city /village/town of residence, or geography of the location [14]. Apps that connect you instantaneously to a doctor as per your need, software that allows the making and maintaining of electronic patient case records, facilitating access for both patients and physicians, android applications that allow you to monitor your vitals and provide timely tips to manage your lifestyle, and many more such up and coming software or electronic programs form the umbrella of population health tools [15].

Population health management (PHM) implies (quality-care-cost) triple objectives of cutting down per capita health expenditure, enriching the health & well-being of the populace, and enhancing every individual’s experience of healthcare [15]. To bring these objectives to fruition, population health management involves bettering access to healthcare for all and fortifying uniform availability of disease screening/ preventive services, and spurring positive behavioral changes [16]. PHM models need hand-in-hand collaboration of specialists, clinicians, nursing staff, health workers, medico-social workers, and active involvement of the family of the patient. Population health management systems when came into the purview of healthcare technology brought about the development of numerous online platforms like risk stratification tools, population health intelligence platform (PHIP), medical management system (MMS), NextGen software, Risk-stratification tools, etc. The functions of these PHM models range from simple access to patient data from varying sources to creating personalized care plans for patients, categorizing patients according to their population according to their health risks, tracking morbidity profiles, analyzing hospitalization trends, aiding patient follow-ups, flagging high-risk patients to even predicting epidemic trends [1].

Electronic health records

Electronic health records (EHR) are a result of a coalition of information technology and telecommunication methods with population health management aims. Also called electronic medical records (EMR), EHRs support computerized clinical decision support systems and facilitate the patient taking active responsibility for their healthcare, which is vital for improved prognosis in non-communicable diseases like diabetes and cardiovascular disorders whose management needs stringent participation of the patients too [17]. As these systems collect an increasing amount of patient data over time, EMRs become the base for more accurate predictions and more appropriate tools to aid medical personnel with efficacious patient health management [18]. The WHO Director-general in 2018 stated that while AI in health is facilitating our disease surveillance, EHRs are a must in establishing continuum of care [19]. EHRs are a set part of health systems of many developed nations like the USA, Great Britain, South Korea, Denmark, Japan, and Israel but in some countries like Brazil, China, India, and Canada are facing roadblocks like non-integration of EHRs, lack of universal patient identifier, slow uptake of EHRs, and absence of national strategy regarding EHRs [20].

Healthcare applications

In recent years smartphone applications have gained a lot of popularity, the reason being the easy accessibility, cost-effectiveness, and reasonable customer satisfaction. Healthcare professionals and the medical field have used this means of technology to reach the community. Areas like lifestyle modification, diet, exercise, yoga, meditation for mental health, and many more have been covered. Most of these apps are free, easy to use, and can be downloaded for online or offline use. A highly elaborative digitally evaluated data is calculated for almost all daily activities; calories consumed, steps walked per day, vital heart rate, and pulse are also traced. There are digital fitness wearables available that provide us with our day-to-day health matrix on our smartphones [21].

Frequent updates on our vitals, physical fitness, and calorie intake help us in being mindful of our health. With advancing technology, one might be able to seek prompt medical attention whenever any disturbance is identified by these personal health status trackers. Fitbit wearables are among the rising range of personal health monitoring devices which allow such health status monitoring. WebMD, a highly recognized website that leads the pack of websites in providing possible underlying causes for symptoms experienced, has also recently migrated as a successful mobile phone app [22]. The number of people using digital platforms is exponentially exceeding every year. In 2017, an insurance company reported online and telephone consultations got more popularized than traditional face-to-face consultations [23]. 

Disease reporting & e-epidemiology

Last defined epidemiology as "the study of distribution and determinants of health-related events in specified populations and application of this study for prevention and control of health-related events” [24]. E-epidemiology uses communication technology and electronic media to hasten the process of collecting and refining relevant data related to disease incidents or outbreaks and directs which preventive measures to tackle the outbreak [25]. Robust data lays the ground for an impactful preventive strategy. E-epidemiology gets us a nearly up-to-date progress sheet of the disease and assists in planning an efficient plan for the distribution of health task force as needed [26].

Data mining

It is now an established belief in the healthcare industry that healthcare data is surging across the globe, but despite this escalating pool of data, the extent of its use in practice is currently very minimal [27,28]. Data mining, a subdivision of AI utilizes extensive amounts of data to derive new, relevant, notable information and disease patterns, thus laying the ground for better and advanced clinical management [28]. In the last decade, there have been many studies exploring the uses of data mining techniques in healthcare like an Iranian study of data mining approach to find determinants of length of hospital stay [29], an Italian study with a data mining approach to classifying Parkinsonism by gait [30], and many more. Additionally, by using data mining concepts and techniques, health systems can array together variables with similar characteristics and forecast probable health events. A rightful cause for concern in this sea of benefits is the breach of privacy or the leak of personal information from health information systems. So, the challenge now is to safeguard this sensitive data without dampening the utility of data mining processes [31].

Health information and news reporting

Not long back health emergencies were reported in the newspaper, on the radio, or on television, when people had to wait for it to be broadcasted, which also created a time lag between the time of news received and the real situation going on. Today, thanks to communication technologies, reporting of health information happens almost instantaneously over multiple mediums like the internet, social media, and other broadcasting outlets [32]. A case in point is Arogya Setu, an app developed in India during the recent pandemic to help civilians self-assess their COVID-risk status and provide best practices and care advice accordingly. This app was introduced much before the vaccines were ready and played a vital role in keeping up with outbreaks in a population of over 1.3 billion [33].

Social media

This is the era of social media. Popular social media platforms like Facebook and YouTube play a key role in providing new health-related information to the general public. A report suggests that in total 64% of information reaching the public can be attributed to YouTube alone [34]. Now, if we were to add all social media platform numbers to this, we can truly appreciate the amount of impact these sites have in getting information out in world space. Vice-versa, social media sites are a viable option to collect data or news from the general public in times of an outbreak or lockdown. These outlets allow instant and quick access to the latest information. Furthermore, they make connecting with experts and specialists very feasible and possible.

But these social media platforms come with a big dark side: the lack of authenticity of information floating here. Point in case is of the recent syndemic of COVID-19 and infodemic, when a sea of misinformation masquerading as latest updates in healthcare was disseminated far and wide and caused confusion among the masses and concern among the governments about the potential harm of the infodemic. Research is underway on using data mining techniques to check authenticity and filter misinformation from these platforms [35][36].

Online teaching

Online learning has been a true game changer in recent years. In the medical field, this technological innovation has to the most extent succeeded in breaking down the limitations of distance and lack of expert faculty. Online activities like continuing medical education (CME) and webinars are a key requirement to collect credit points, which allow the renewal of medical licenses in many states of India and abroad. Online CMEs are more feasible for healthcare professionals to attend regularly without stressing about the lack of time and distances to travel for onsite CMEs [37]. A medico distant learning from a village in India can attend the very same lectures as students residing at Harvard Medical School. And this all is possible because of official online portals of education. International health organizations like the United Nations Children's Fund (UNICEF) and Doctors Beyond Borders create awareness and teach the masses about basic health needs like clean drinking water, sanitation, and hygiene through their online platforms [38].

The COVID-19 pandemic caused unprecedented school closures across the world and remote learning options included multimedia and digital platforms (TV, YouTube). According to a survey done by UNICEF on effective remote learning modes in COVID, only 27% of low and lower-middle-income countries had an operational digital learning policy as compared to developed countries and barely 25% of these developing nations ensured free or subsidized internet access in 2021 [39]. According to analyses by the United Nations, close to 50 crores of students faced accessibility issues, three-fourths of which belonged to below-poverty-line families [40].

Barriers to access

Connectivity and Affordability

The prerequisite for good connectivity is everyone having internet and a device to connect to it. In the 1990s, before broadband, the maximum speed of dialups was 56 kbps. Broadband brought in high-speed internet which was further elevated by the development of fiber optic cable and 5G networks [41]. While India ranks second in the world for active network users, internet penetration across India was just 45% in 2021 [42]. This scenario is mirrored in all countries of the world alike. Table 1 depicts this scenario among different world bank regions in the year 2020 [43]. A greater proportion of network providers are private entities, and they usually prefer to set up their services in areas that will be profitable for their businesses. Communities comprising the aging population, low-income groups, or those residing in rural areas are most likely to lose out on good connectivity due to this [44]. Many countries cannot embrace the latest healthcare innovations due to a lack of consistent internet supply [45].

**Table 1:** Population (%) with internet coverage in 2020 Figures for the table taken from: International Telecommunication Union (ITU) World Telecommunication/Information and Communications Technology (ICT) Indicators Database, The World Bank Group, [43]

Country	World Bank Region	Income level as per World Bank knowledge base	% of population with internet connectivity
China	East Asia and the Pacific	Upper middle income	70%
Australia		High income	90%
Tajikistan	Europe and Central Asia	Lower middle income	22%
Germany		High income	90%
Ecuador	Latin America and the Caribbean	Upper middle income	65%
Brazil		Upper middle income	81%
Iraq	The Middle East and North Africa	Upper middle income	60%
Saudi Arabia		High income	98%
Canada	North America	High income	97%
USA		High income	91%
Afghanistan	South Asia	Low income	18%
Maldives		Upper middle income	63%
South Africa	Sub-Saharan Africa	Upper middle income	70%
Uganda		Low income	20%

Health and Digital Literacy

Health literacy is described as the ability to acquire, process, and understand the fundamental healthcare statistics and system and then apply these to make befitting healthcare-related decisions. Along with the technological and financial restraints, health literacy also poses a major setback to accessing online health services. Digital literacy is a closely linked concept to this; it means the capacity to find as well as use digital content and create and share it. Some causes, like access and communication problems, physical and mental restrictions, as well as social factors like age, gender, and literacy, not only drive the digital divide and digital literacy but also health literacy. An individual who has low health literacy tends to be increasingly skeptical about health-related technology and finds himself overloaded by the endless sea of knowledge available. He or she is also anticipated to spend comparatively less time on the web understanding health-related topics along with newer treatment modalities or even creating an account online for accessing medical records [46,47].

Cultural Barriers

Restrictive cultural beliefs, limited internet coverage, and poor health literacy weave together a web of cultural barriers, in which the hardest hit groups are females and children. Females in some places are subject to socio-cultural norms discouraging autonomy and inhibiting them from making finance-related decisions that include buying a mobile or a laptop. According to a 2014 Telecomm Regulatory Authority of India report, barely 30% of the total 900 million-plus cellphone subscribers were women. Citing reasons like a disturbance in schoolwork and fears of adultery, cell phones were banned in a few villages in India [48].

**Figure 2:**
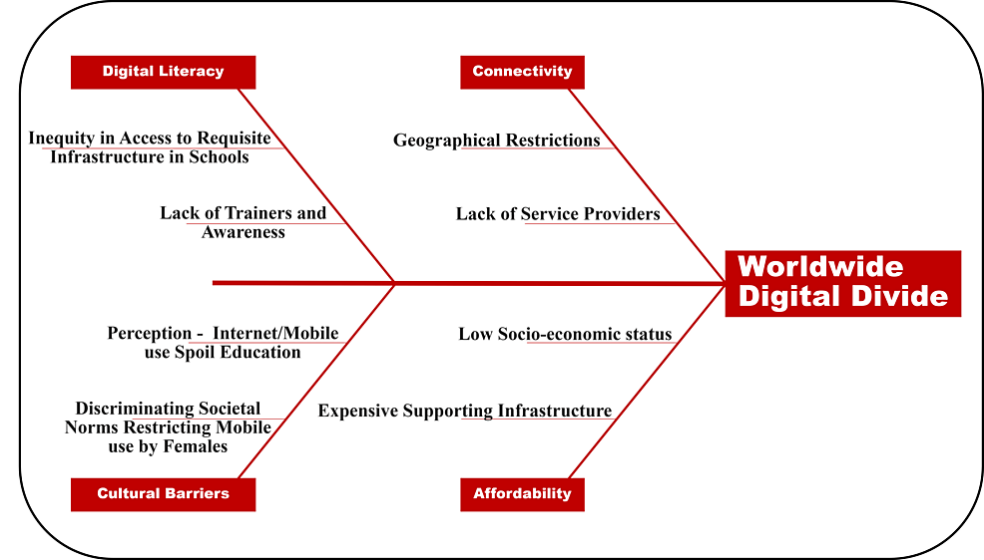
Fish-bone diagram depicting key barriers to the equity in digital access to healthcare globally

About 25 percent of women have lesser internet access than men in developing nations, and this gender gap increases to 45% in Sub-Saharan Africa. Contrarily, net usage by all is promoted in many South Asian countries like South Korea wherein it is considered a literacy aid and a tool to better their future [49,50]. Language proficiency is also a notable barrier, as not all of the content of online courses and e-health apps have been made available in local languages. Figure 2 summarizes the key barriers to equity in digital access to healthcare globally.

Efforts to minimize the digital healthcare divide

Internet is ingrained in almost all walks of our life; be it ease of access to healthcare or information collection. Keeping this in mind, access to the internet has been acknowledged by the United Nations as a basic human right [51]. The prevalent digital divide today can be primarily attributed to affordability and accessibility. Electricity was once upon a time a luxury, not a necessity. Internet usage is going through the same journey now. According to the World Bank, “Over the past five years, there has been a veritable explosion of technology and consumerism in India, but this has occurred largely in urban and middle-class communities” [52]. India has over 500,000 villages scattered over its expanse with over half a billion population lagging in access to basic technology. Nearly two decades have passed since the Universal Service Obligation Fund (USOF) was launched with the aim to provide uniform internet devices to people in rural, remote, and tribal villages of the country [53]. But even today up-to-date knowledge of potential markets and best farming practices remain cloaked from the people who need them the most due to this digital divide.

When the government of India announced a complete lockdown of the nation for three months to curb the spread of COVID, the outpatient department (OPD) services were also shut down. As a countermeasure, immediately after the closure of OPDs, the Ministry of health and family welfare (MoHFW), released India’s first telemedicine guidelines [54]. India still has a long way to go in making this telemedicine initiative uniformly accessible to even the remotest part of the nation and for that, the nation needs remarkable and seamless broadband coverage and a good economy. The situation of this divide is not much different in developed countries either. In the United States of America (USA), the efforts of the Universal Service Fund and the Department of Agriculture’s Rural Utility Service program are in the works to establish web services throughout the country [55]. ConnectHome, an initiative by the President of the USA in 2015, aimed to assist children from low socioeconomic homes with internet access. The purpose was to resolve the problem of lack of network hampering students’ ability to do their schoolwork. In the last 6 years, 37% of target children have gained access through ConnectHome. ConnectHome also improved the ability of caregivers at home to provide proper services to the patient under their care through their online resources [56].

The challenge in Africa is its expanse of boundless deserts, impenetrable jungles, and a plethora of rural and tribal areas where the homes do not yet have electrical grids [57]. Organizations such as eHealth Africa are toiling to provide online health services to neglected populations. Telemedicine Africa is a network of healthcare professionals that are committed to providing online consultations to any citizens in need in South Africa. Rural areas have referral centers that can connect to a Virtual Telehealth network provided the internet is stable and gain access to basic healthcare consultations and wellness practices [58].

The way forward

The testament of the United Nations declaring internet access as a basic right only nailed the realization among the ruling bodies of the entire world that equitable digital access is a necessity [51]. The COVID-19 pandemic expounded on that and has made uniform high-speed internet a desirable goal for all nations so that healthcare is not denied to its citizens because of unforeseen circumstances. But for developing nations with varied geography of hilly areas, remote islands, and tribal pockets in deep forests, the goal is daunting, not considering the cost of this ambitious developmental milestone. It is an established fact in India that even the villagers who don’t have access to a toilet have a smartphone [59]. And while teleservice providers have made affordable high-speed internet a way of life for individuals globally, internet coverage in remote and rural areas is still an ongoing challenge. Nguyen et al. showed that while age, race, and poverty acted as barriers, increasing digital literacy and health awareness levels were positively associated with the uptake of digital health [44].

The solution may lie in the technology itself that we are trying to make accessible. Further efforts are needed to minimize the cost of scaling up the technology, by developing modalities that are feasible, affordable, and acceptable to the people and the community. Moreover, a supportive environment to nurture research and development in developing countries might require intervention at the macro-level in terms of policy-making, and effective implementation mechanisms by promoting intersectoral coordination and at the micro-level by the inclusion of a broader stakeholder perspective. This is essential because the success of technology in healthcare depends not only on its application but also on the availability of adequate infrastructure to take it to the grass root level beneficiary.

## Conclusions

There is a digital divide in existence today; health care technology is advancing but access is not equitable yet. Nevertheless, all the nations of the world alike are in consensus to keep the research and efforts ongoing to bridge this divide and make technology and healthcare more accessible to every man and woman. A blend of contextualization of the technology for the beneficiaries it affects and macro-level policy decisions may help in narrowing the existing digital divide.
